# Involvement of oxidative stress in orofacial mechanical pain hypersensitivity following neonatal maternal separation in rats

**DOI:** 10.1038/s41598-023-50116-1

**Published:** 2023-12-20

**Authors:** Chihiro Soma, Suzuro Hitomi, Eri Oshima, Yoshinori Hayashi, Kumi Soma, Ikuko Shibuta, Yoshiyuki Tsuboi, Tetsuo Shirakawa, Takashi Kikuiri, Koichi Iwata, Masamichi Shinoda

**Affiliations:** 1 Department of Pediatric Dentistry, Nihon University School of Dentistry, 1-8-13 Kandasurugadai, Chiyoda-ku, Tokyo, 101-8310 Japan; 2 Department of Physiology, Nihon University School of Dentistry, 1-8-13 Kandasurugadai, Chiyoda-ku, Tokyo, 101-8310 Japan; 3 Department of Oral and Maxillofacial Surgery, Showa University School of Dentistry, 1-5-8 Hatanodai, Shinagawa-ku, Tokyo, 142-8555 Japan

**Keywords:** Neuroscience, Physiology

## Abstract

Patients with persistent pain have sometimes history of physical abuse or neglect during infancy. However, the pathogenic mechanisms underlying orofacial pain hypersensitivity associated with early-life stress remain unclear. The present study focused on oxidative stress and investigated its role in pain hypersensitivity in adulthood following early-life stress. To establish an early-life stress model, neonatal pups were separated with their mother in isolated cages for 2 weeks. The mechanical head-withdrawal threshold (MHWT) in the whisker pad skin of rats received maternal separation (MS) was lower than that of non-MS rats at postnatal week 7. In MS rats, the expression of 8-hydroxy-deoxyguanosine, a marker of DNA oxidative damage, was enhanced, and plasma antioxidant capacity, but not mitochondrial complex I activity, decreased compared with that in non-MS rats. Reactive oxygen species (ROS) inactivation and ROS-sensitive transient receptor potential ankyrin 1 (TRPA1) antagonism in the whisker pad skin at week 7 suppressed the decrease of MHWT. Corticosterone levels on day 14 increased in MS rats. Corticosterone receptor antagonism during MS periods suppressed the reduction in antioxidant capacity and MHWT. The findings suggest that early-life stress potentially induces orofacial mechanical pain hypersensitivity via peripheral nociceptor TRPA1 hyperactivation induced by oxidative stress in the orofacial region.

## Introduction

During the neonatal period, psychological and physical stress due to parental ignorance and maltreatment occasionally leads to chronic pain later in life^1,2^. Patients with chronic pain are reportedly 3.75 times more likely to have experienced early-life stress^2^. On exposure to acute stress, the corticotropin-releasing factor is released from the hypothalamus, and it causes the release of adrenocorticotropic hormone, resulting in the release of glucocorticoids (GCs) from the adrenal cortex. Subsequently, the activity of the hypothalamic–pituitary–adrenal (HPA) axis is suppressed by negative feedback^3^. Early-life stress alters the HPA axis and interferes with the regulatory function of hormonal balance or stress responses, which are implicated in the pathogenesis of chronic pain^3,4^. Therefore, psychological experiences, such as early-life stress, can influence the development of the nociceptive system.

Some pathogenic mechanisms underlying pain hypersensitivity in adults following early-life stress have been reported in animal studies. In the central nervous system, dysfunctions of the descending modulation of pain and neuroinflammation in the hippocampus and hypothalamus have been observed in maternally separated (MS) rodents^5,6^. In the periphery, the excitability of primary neurons is potentiated by an increase in Nav1.8 expression and a decrease in voltage-gated potassium channel 1.2 expression in the dorsal root ganglion neurons of MS rats^7,8^. Previously, we reported that the number of P2X purinoceptor 3 (P2X_3_)-expressed trigeminal ganglion neurons increases due to MS-induced enhancement of neonatal corticosterone (CORT) signaling, causing orofacial mechanical allodynia in adulthood^9^.

Oxidative stress is the imbalance between oxidant and antioxidant production in cells and/or the body. Oxidants, such as reactive oxygen species (ROS), are highly reactive chemicals formed from diatomic oxygen. Antioxidant enzymes, such as catalase and superoxide dismutase, and antioxidant substances, such as glutathione, inhibit ROS oxidation. If oxidant production exceeds their elimination by antioxidants, oxidative stress occurs^10^. Pain-related transient receptor potential (TRP) channels have recently been reported to be sensitive to oxidants^11,12^. In particular, among TRP channels, the TRP ankyrin 1 (TRPA1) channel is known to be most sensitive to oxidants^13^. TRPA1 is a nonselective cation channel expressed in fibroblasts, sensory neurons, epithelial cells, and keratinocytes and is involved in pain^14,15^. ROS can activate TRPA1 by oxidizing cysteine residues at its cytoplasmic N-terminal, leading to pain hypersensitivity in rodents^12,16^. A recent study indicated that oxidative stress is increased by a decrease in the antioxidant enzyme catalase in the hippocampus owing to early-life stress in adult mice^17^. Therefore, oxidative stress is involved in early-life stress-induced pain hypersensitivity in adults. However, the oxidative stress-related pathogenic mechanism underlying orofacial pain hypersensitivity associated with early-life stress remains unclear.

Therefore, the present study aimed to examine the oxidative stress-related pathogenetic mechanism underlying early-life stress-induced orofacial pain hypersensitivity in adults. MS rats constituted an early-life stress model. First, this study investigated the change in mechanical pain sensitivity in the whisker pad skin following MS. Thereafter, post-MS oxidative damage to the whisker pad skin, antioxidant capacity, and plasma CORT levels were determined. Finally, the effects of ROS scavenging as well as TRPA1 and CORT receptor antagonism on orofacial mechanical pain hypersensitivity were investigated.

## Results

### MS causes mechanical pain hypersensitivity in adulthood

**Figure 1:**
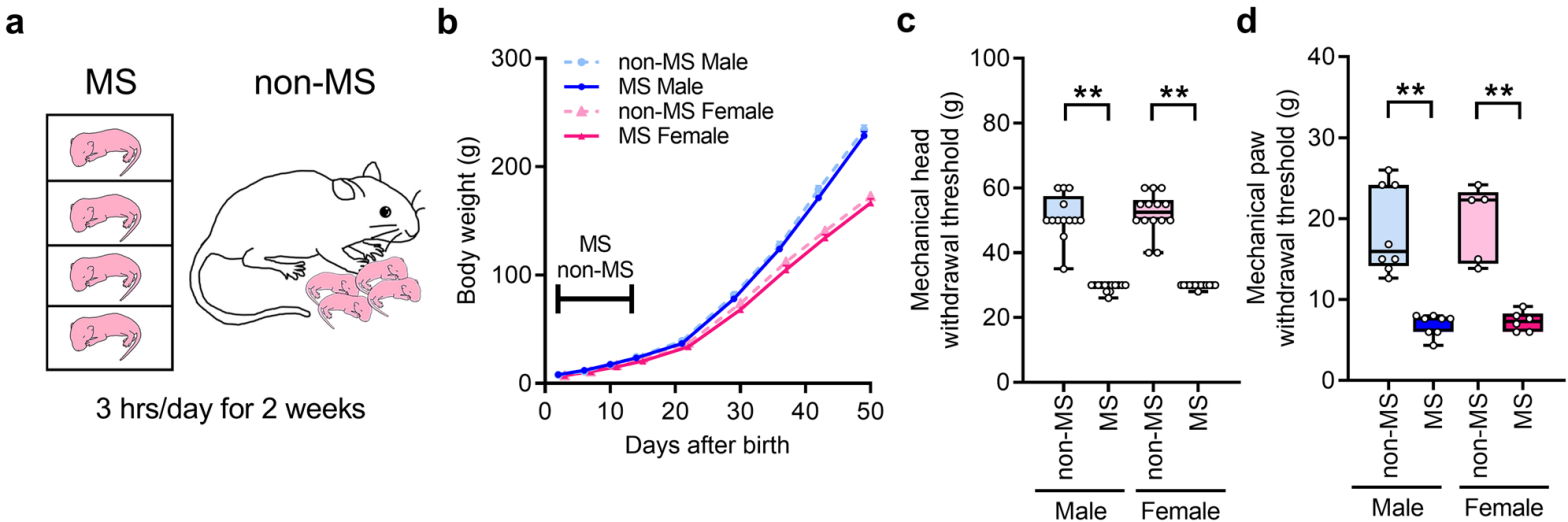
Schematic image of maternal separation (MS) and change in body weight and mechanical withdrawal threshold. (**a**) An illustration of the MS and control (non-MS) rats. (**b**) Change in body weight (n = 9 in each group). (**c**) Mechanical head-withdrawal threshold in the whisker pad skin of non-MS and MS rats at 7 weeks (n = 13–14 in each group). **P < 0.01, Kruskal–Wallis test followed by Dunn’s multiple comparisons test. (**d**) Mechanical paw-withdrawal threshold in the hind paws of non-MS and MS rats at 7 weeks (n = 5–8 in each group). **P < 0.01, Kruskal–Wallis test followed by Dunn’s multiple comparisons test.

Some littermates were separated from their mothers for 14 days (MS rats), while the remaining littermates were designated non-MS rats (Fig. 1a). No difference in body weight was noted between male and female MS rats (Fig. 1b). The head-withdrawal threshold for mechanical stimulation of the whisker pad skin was significantly lower in MS rats than in non-MS rats (Fig. 1c). The paw-withdrawal threshold for mechanical stimulation of the hind paw was also significantly reduced in MS rats compared with that in non-MS rats (Fig. 1d). In non-MS and MS rats, there were no differences in the mechanical withdrawal threshold in the whisker pad skin and the hind paw between male and female (Fig. 1cd).

### MS increases oxidative stress in adulthood

**Figure 2:**
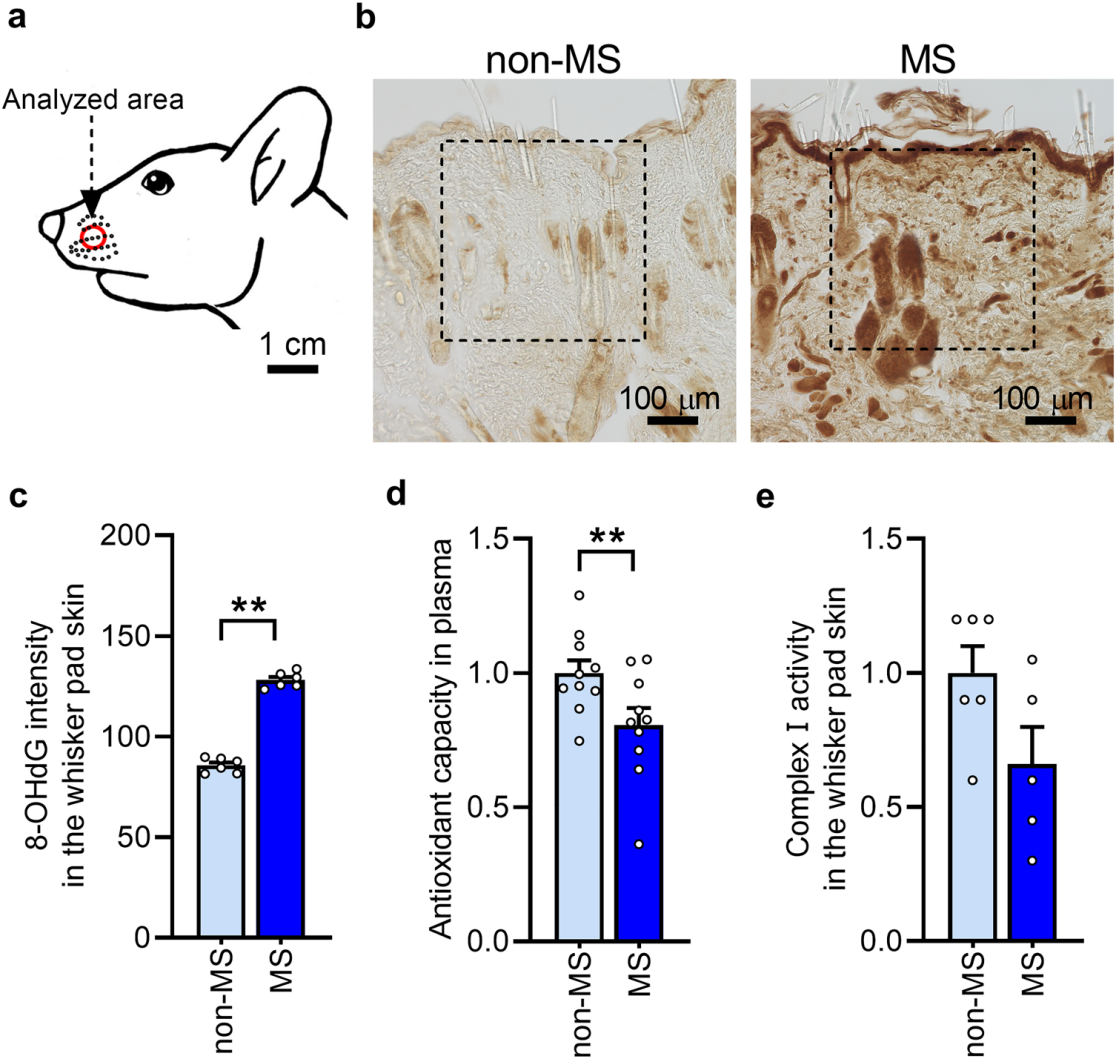
Oxidative stress in the whisker pad skin following maternal separation (MS). (**a**) Analyzed area of the whisker pad skin. (**b**) Microphotograph images of representative 8-hydroxy-deoxyguanosine (8-OHdG) immunoreactivity in the whisker pad skin of non-MS and MS rats at 7 weeks. Dotted square in the images indicate analyzed region. Scale bars indicate 100 µm. (**c**) 8-OHdG intensity in the whisker pad skin (n = 6 in each). **P < 0.01, unpaired *t*-test. (**d**) Plasma antioxidant capacity of MS and non-MS rats (n = 10 in each group). **P < 0.01, unpaired *t*-test. (**e**) Complex I activity in the whisker pad skin of MS and non-MS rats (n = 5–6 in each group). *P* = 0.07, unpaired *t*-test.

To determine whether MS causes excessive oxidative stress, the expression of 8-hydroxy-deoxyguanosine (8-OHdG), a marker of DNA oxidative damage, was initially analyzed in the whisker pad skin (Fig. 2a). Compared with that in non-MS rats, 8-OHdG immunoreactivity was enhanced in MS rats (Fig. 2b). The mean 8-OHdG intensity in the defined area in the MS rats were significantly increased compared with that in the non-MS rats (Fig. 2c). Thereafter, to determine why oxidative stress increased at 7 weeks in MS rats, the activity of complex I of the electron transport chain in the mitochondria and plasma antioxidant capacity were analyzed in the whisker pad skin. Complex I is the primary site of ROS production in the mitochondria^18^. In MS rats, antioxidant capacity was significantly lower than that in non-MS rats (Fig. 2d). Complex I activity tended to be lower in MS rats than in non-MS rats (Fig. 2e).

### Effects of ROS and TRPA1 inhibition in the whisker pad skin on MS-induced orofacial mechanical pain hypersensitivity

**Figure 3:**
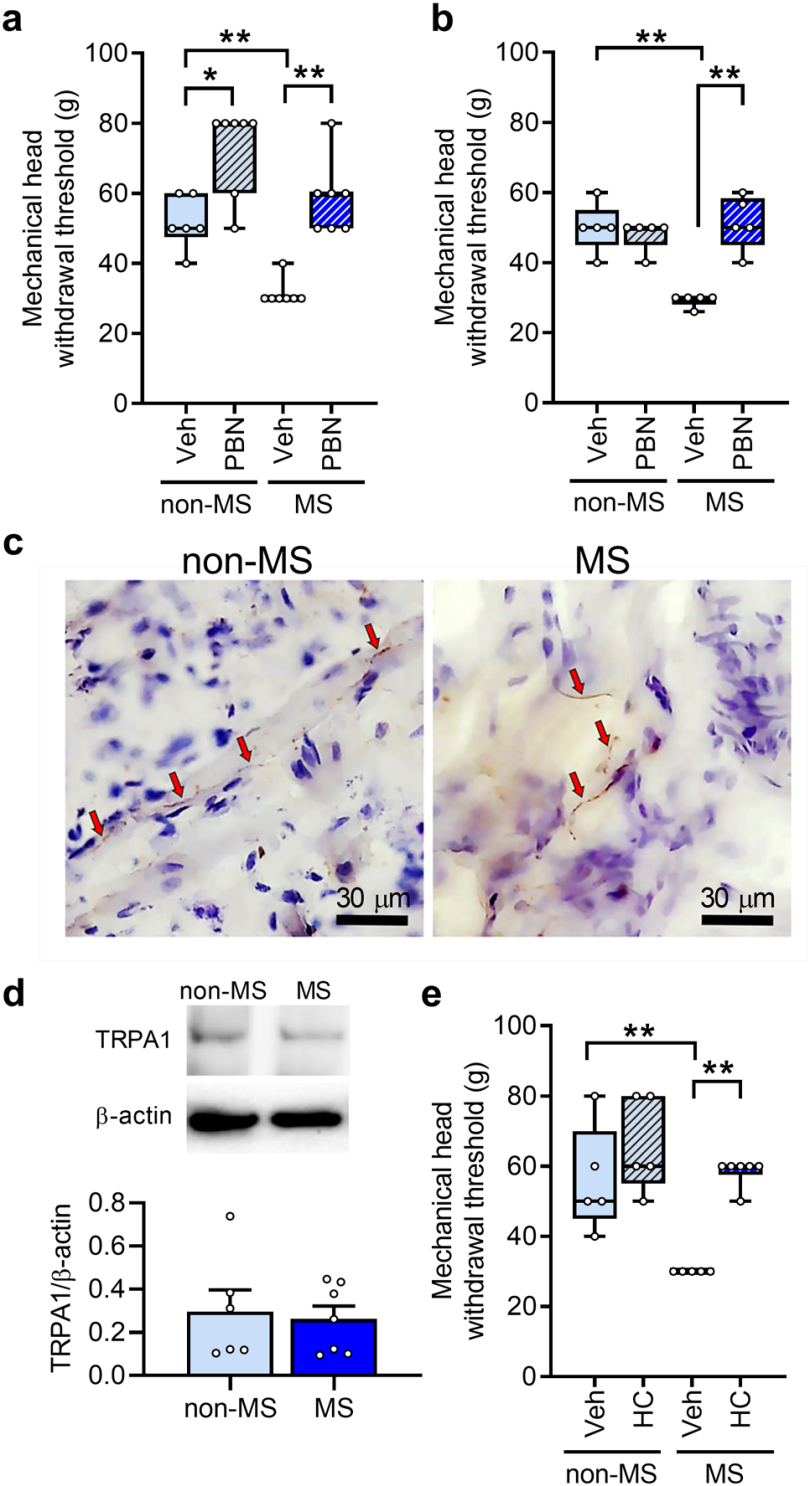
Involvement of reactive oxygen species (ROS) in mechanical pain hypersensitivity following maternal separation (MS). (**a**, **b**) Mechanical head-withdrawal threshold in the whisker pad skin following intraperitoneal (**a**) or subcutaneous (**b**) administration of the ROS scavenger N-tert-butyl-α-phenylnitrone (PBN) in non-MS and MS rats at 7 weeks (n = 6–7 in each group). *P < 0.05, **P < 0.01, Mann–Whitney U test. (**c**) Expression of transient receptor potential ankyrin 1 (TRPA1) in the whisker pad skin in the non-MS and MS rats at 7 weeks. Red arrows denote TRPA1-immunoreactivity. Scale bars indicate 30 µm. (**d**) Immunoblot for TRPA1 in the whisker pad skin at 7 weeks in the non-MS and MS rats (n = 6–7). *P* = 0.77, unpaired *t* test. (**e**) Mechanical head-withdrawal threshold in the whisker pad skin 30 min after intraperitoneal administration of the TRPA1 channel antagonist HC-030031 (HC) to non-MS and MS rats at 7 weeks (n = 5–6 in each group). **P < 0.01, Mann–Whitney U test.

In MS rats, the mechanical head-withdrawal threshold was significantly lower than that in non-MS rats (Fig. 3a). Intraperitoneal administration of the ROS scavenger N-tert-butyl-α-phenylnitrone (PBN) inhibited the MS-induced decrease in the mechanical head-withdrawal threshold. The administration also increased the mechanical head-withdrawal threshold in non-MS rats, suggesting involvement of ROS in mechanical sensitivity under normal conditions. The MS-induced decrease in the mechanical head-withdrawal threshold was also inhibited by subcutaneous PBN administration to the whisker pad skin (Fig. 3b). Subsequently, the TRPA1 channel, which is known to be sensitive to ROS, was expressed in the nerve endings of the whisker pad skin in both non-MS and MS rats^12^ (Fig. 3c). The amount of TRPA1 was not significantly different between the non-MS and MS rats at 7 weeks (Fig. 3d). To ascertain whether TRPA1 was involved in the MS-induced decrease in the mechanical head-withdrawal threshold, the TRPA1 antagonist HC-030031 (HC) was administered to the whisker pad skin, and the mechanical head-withdrawal threshold was measured. In MS rats, the mechanical head-withdrawal threshold was significantly lower than that in non-MS rats (Fig. 3e). The decreased mechanical head-withdrawal threshold recovered to non-MS levels after HC administration to the whisker pad skin (Fig. 3e).

### Involvement of CORT increases during the neonatal MS period in mechanical pain hypersensitivity

**Figure 4:**
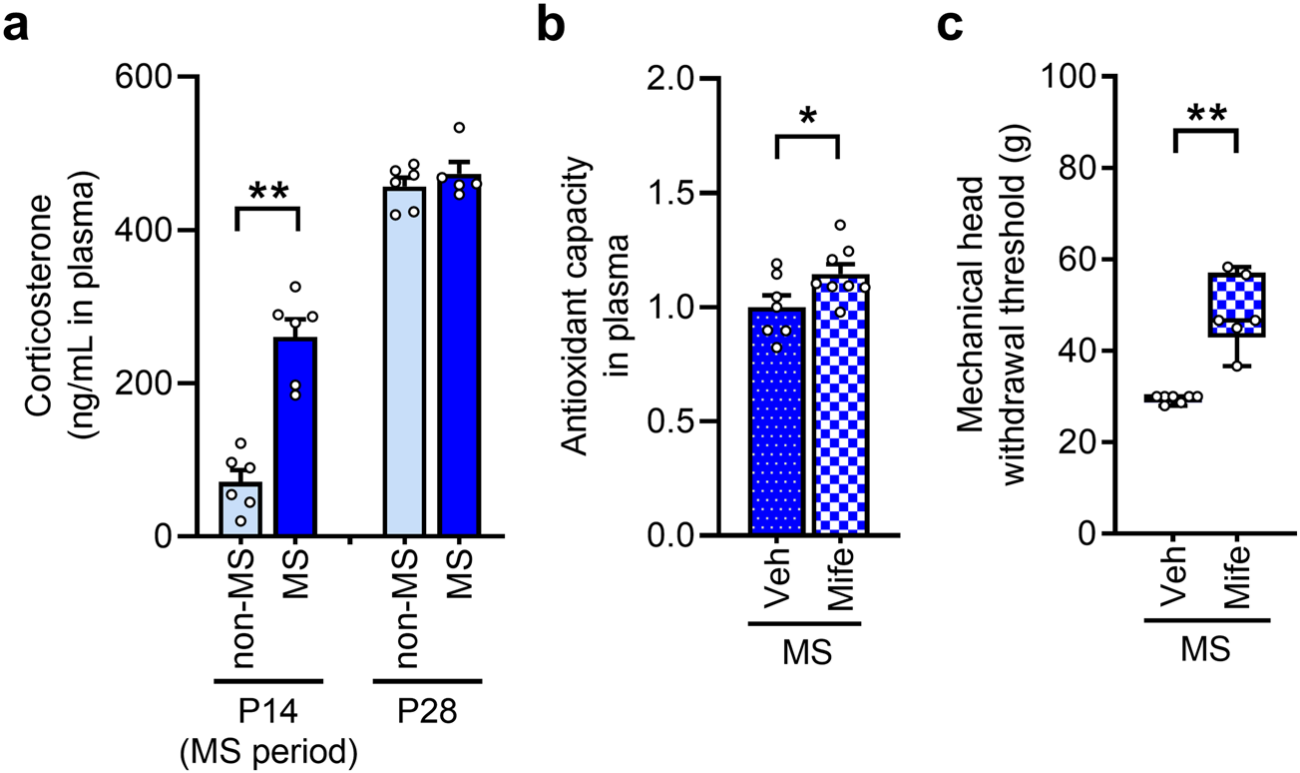
Involvement of corticosterone in decreasing antioxidant capacity and mechanical pain hypersensitivity in maternal separation (MS) rats. (**a**) Concentration of corticosterone in plasma of the non-MS and MS rats at 14 and 28 days (n = 5–6 in each group). **P < 0.01, unpaired *t*-test. (**b**) Antioxidant capacity in plasma following subcutaneous administration of the CORT receptor antagonist mifepristone (Mife) during MS from day 2 to day 14 (n = 7–8 in each group). *P < 0.05, unpaired *t*-test. (**c**) Mechanical head-withdrawal threshold in the whisker pad skin following subcutaneous administration of Mife (n = 6–7 in each group). **P < 0.01, Mann–Whitney U test.

The plasma concentration of CORT on postnatal day 14 (P14) was significantly higher in MS rats than in non-MS rats. On day 28, this concentration increased; however, no difference was observed between non-MS and MS rats (Fig. 4a). To examine the effect of CORT increase during the MS period on the decrease in antioxidant capacity and the mechanical head-withdrawal threshold, mifepristone, a CORT receptor antagonist, was administered during this period, and antioxidant capacity and the mechanical head-withdrawal threshold were subsequently measured. The plasma antioxidant capacity at week 7 was increased by mifepristone administration in MS rats (Fig. 4b). Mifepristone administration inhibited the decrease of the mechanical head-withdrawal threshold in MS rats (Fig. 4c).

## Discussion

In the present study, early-life stress caused by 2-week MS resulted in mechanical pain hypersensitivity in the whisker pad skin and hind paw later in life. The 8-OHdG intensity in the whisker pad skin were increased in MS rats. Plasma antioxidant capacity and complex I activity in the whisker pad skin decreased in MS rats. Orofacial mechanical pain hypersensitivity in MS rats was inhibited by the subcutaneous administration of PBN, an ROS scavenger, or TRPA1 antagonist to the whisker pad skin, indicating that MS-induced oxidative stress contributed to mechanical pain hypersensitivity. On days 14 and 28, the concentration of CORT increased in MS rats. Blockage of CORT signaling during MS alleviated the decrease in antioxidant capacity and mechanical pain hypersensitivity in adult rats. These results suggest that MS-induced orofacial mechanical pain hypersensitivity emanates from TRPA1 activation, which is caused by MS-induced ROS, owing to the low antioxidant capacity of the whisker pad skin.

In our study, TRPA1 blockage inhibited the decrease in the mechanical head-withdrawal threshold in the whisker pad skin of MS rats. TRPA1 is a nonselective cation channel that is activated by various pungent compounds and noxious chemical, mechanical, and cold stimuli^12,19^. In addition, TRPA1 can be activated by the oxidation of cysteine residues in the cytoplasmic N-terminals of TRPA1, leading to pain hypersensitivity in rodents^12,16^. As oxidative stress increased in the whisker pad skin of MS rats, the increased ROS potentially activate TRPA1. TRPA1 is expressed in fibroblasts, sensory neurons, epithelial cells, and keratinocytes^14,15^. Fibers that were likely to be nerve endings expressed TRPA1 in the whisker pad skin. Together with the suppression of mechanical pain hypersensitivity by PBN administration, ROS–TRPA1 signaling in peripheral tissues is probably involved in mechanical pain hypersensitivity in MS rats. Mechanical pain hypersensitivity was also found to be induced in MS rats’ hind paws, suggesting that mechanical pain hypersensitivity via ROS–TRPA1 signaling develops throughout the entire body. Several previous studies have used 8-OHdG as a marker of free radical-induced nucleoside oxidation in DNA and mitochondrial DNA^16,20^. In general, antioxidants can eliminate oxidants over a brief period. For example, the increased 8-OHdG levels caused by exposure to high-dose ionized radiation were reduced within 24 h of irradiation in mice^21^. In the present study, 8-OHdG immunoreactivity increased in the whisker pad skin at 7 weeks in MS rats, suggesting that oxidative stress caused by early-life stress continues into adulthood. Complex I is a vast enzyme complex in the mitochondrial electron transport chain and is the primary site of ROS production in mitochondria^18^. In week 7, complex I activity in the mitochondria decreased, though not significantly, in MS rats compared with that in non-MS rats, indicating that mitochondrial function is probably not adequately disturbed by MS. Conversely, antioxidant capacity was significantly decreased in MS rats. These results suggest that early-life stress-induced oxidative stress is caused by reduced plasma antioxidant capacity but not by increased ROS production in adults. In the present study investigated oxidative DNA by assessing 8-OHdG expression, but not by evaluating the ROS level directly. In addition, time-course alterations in 8-OHdG expression and antioxidant capacity were not analyzed. Further experiments are required to clarify ROS dynamics following early-life stress.

GCs are secreted from the adrenal cortex in response to stress^22^. Our results revealed that the concentration of CORT, which is a rodent GC, increased during the MS period and was at the same level as that in non-MS rats 2 weeks after MS, indicating CORT overproduction during stress loading. This study also demonstrated that mechanical pain hypersensitivity was inhibited by CORT signaling blockage during the MS period. Furthermore, the blockade recovered antioxidant capacity at 7 weeks. These results suggest that CORT overproduction during MS is involved in the decrease in antioxidant capacity in adults, leading to the continuous activation of TRPA1 by excess ROS, which causes mechanical pain hypersensitivity in MS rats. Since early-life stress alters the HPA axis^3^, a connection probably exists between the HPA axis and antioxidant capacity. The detailed mechanisms will be examined in future studies.

The present study addressed mechanical pain hypersensitivity following MS. Other mechanisms that induce hypersensitivity possibly exist. In the central nervous system, K^+^–Cl^−^ cotransporter (KCC2) regulates intracellular chloride levels by exporting Cl^−^ from the extracellular environment^23^. KCC2 is expressed at low levels during the neonatal period but is developmentally upregulated and responsible for the GABAergic switch, which is a functional excitatory-to-inhibitory transition^23–25^. Early-life stress has recently been demonstrated to inhibit the expression of KCC2, leading to a delay in the GABAergic switch^25^. These results indicate that early-life stress-induced KCC2 downregulation renders GABAergic signaling excitatory, leading to pain hypersensitivity. Furthermore, the expression of the adenosine triphosphate receptor P2X is involved in post-MS pain hypersensitivity^26,27^. P2X receptors are involved in nociceptive signal transduction^28^. Visceral hypersensitivity is inhibited by P2X_4_ blockage in the L4–L5 spinal cord region of MS rats via brain-derived neurotrophic factor signaling^27^. In our previous study, the number of P2X_3_-positive trigeminal ganglion neurons increased later in life following MS. This increase was inhibited by CORT receptor antagonism during the MS period, indicating that P2X_3_ was increased by the enhancement of CORT signaling^26^. In addition, P2X_3_ antagonism suppresses MS-induced mechanical pain hypersensitivity^26^. These mechanisms are potentially involved in MS-induced pain hypersensitivity. Some previous studies have reported that glucose starvation causes oxidative stress^29,30^. In the present study, because neonatal rats were prevented from breastfeeding during MS period, MS rats were potentially received starvation stress. However, since no difference of body weight was observed between non-MS and MS rats, the possibilities of starvation-induced oxidative stress is low.

In a previous animal study, the mitochondrial oxidative stress levels assessed via 8-OHdG were higher in males than in females owing to lower antioxidant production in males than in females^31^. Hence, oxidative stress-induced pain hypersensitivity is expected to be higher in male than in female. However, the present study found that both male and female rats induced mechanical pain hypersensitivity following MS. Possibly, the present MS treatment produced sufficient ROS which cannot be delated by antioxidants even in female rats. In female rats, low oxidant production and high antioxidant levels have been found to be cancelled by ovariectomy^31^, suggesting that estrogen is strongly associated with oxidative stress regulation. Since early life stress including MS potentially alters estrogen levels^32^, no difference in the mechanical head-withdrawal threshold were observed between male and female rats in the present study. In contrast, a previous study has reported that pain sensitivity in adult has been decrease in male rats but not in female rats following early life stress due to the difference in hormonal balance including estrogen and progesterone^33^. However, no sex differences were observed in biochemical and molecular tests in the study. Further studies are needed to determine the MS-induced pain mechanism in female rats.

In conclusion, the present study demonstrated that early-life stress causes low antioxidant capacity later in life, leading to excess ROS production. Subsequently, ROS activate and sensitize TRPA1 in nerve endings, causing mechanical pain hypersensitivity. MS-induced early-life stress is considered to reflect infant neglect or abuse in humans. Neglect in children has occasionally leads to undernutrition^34^. The improvement of undernutrition via sufficient nutrient intake, including that of antioxidants such as vitamin C, polyphenols, and beta-carotene, can mitigate oxidative stress, leading to the alleviation of early-life stress-induced mechanical pain hypersensitivity^35^. Therefore, childcare during development is critical for healthy growth.

## Methods

### Animals

Pups from timed-pregnant Sprague–Dawley rats (n = 192; 23 pregnant rats, 141 male pups, 28 female pups; Japan SLC, Hamamatsu, Japan) were used in this study. The details in animal section can be found as Supplementary Table S1. The dams were housed in individual cages, maintained on a 12/12-h light–dark cycle (lights on from 7:00 a.m. to 7:00 p.m.) in a temperature-controlled environment, and provided food and water ad libitum. The Animal Experimentation Committee of Nihon University approved all experiments (animal protocol numbers: AP20DEN007 and AP22DEN026), and the procedures were performed according to the guidelines of the International Association for the Study of Pain^36^. The study was performed in compliance with ARRIVE guidelines. The number of animals used and degree of animal suffering were maximally reduced in all experiments.

### Maternal separation (MS)

From P2 to P14, MS was performed as described previously^26^. After determining their sex, the pups were divided into two groups (MS and non-MS) by marking their ears. MS group pups were placed in isolated cages daily for 180 min and kept in a temperature-controlled environment at 22 ± 2 °C. After the separation period, the pups were returned to their maternity cages. Non-MS pups were left with their respective dams. On a postnatal day 21, the pups were weaned and separated with their mothers in the different cage. All pups were weighed weekly.

### Measurement of the withdrawal thresholds of the whisker pad skin and hind paw in response to mechanical stimulation

To measure the mechanical head-withdrawal threshold of the whisker pad skin, rats were trained to keep their snouts protruding from a plastic cage through a small hole in its front wall. They were allowed to escape freely from the applied stimulation. After successful training, mechanical stimuli (4, 6, 8, 15, 26, 30, 40, 50, 60, and 80 g) were applied to the skin on the left whisker pad using von Frey filaments. To measure the mechanical withdrawal threshold in the hind paw, rats were acclimated in a clear plastic cage (19 × 21 × 15 cm) using wire-netting. After acclimation, the mechanical withdrawal threshold was measured through wire netting using a set of von Frey filaments used for the whisker pad skin. The intensity of the minimum pressure that evoked a withdrawal response for more than three of the five stimulations was determined as each mechanical threshold. All behavioral tests were conducted under blinded conditions.

### Drug administration

The nonspecific free radical scavenger PBN (100 mg/kg; Sigma-Aldrich, St. Louis, MO, USA) was intraperitoneally administered at 7 weeks. The PBN (500 μg/ 50 μL/ rat) and TRPA1-selective antagonist HC-030031 (125 μg/ 50 μL/ rat; Wako, Osaka, Japan) were also subcutaneously administered to the left whisker pad skin at week 7. The CORT receptor antagonist mifepristone (Mife; 5 μg/μL in 50% ethanol, 20 μL on P2–P6, 30 μL on P7–P9, and 35 μL on P10–P14; RU486, Sigma-Aldrich) or vehicle (50% ethanol) was subcutaneously administered daily to MS rats on P2–P14. Drug concentrations were determined based on previous studies^16,26^.

### Tissue and blood sample collection

Whisker pad skin tissues were extracted from MS and non-MS rats using a 6-mm-diameter sterile disposable biopsy punch (Kai Medical, Solingen, Germany) at 7 weeks under deep anesthesia with inhalation of isoflurane (3%; Mylan, Canonsburg, PA, USA) and intraperitoneal administration of butorphanol (2.5 mg/kg; Meiji Seika Pharmaceutical, Tokyo, Japan), medetomidine (0.375 mg/kg; Xenoac, Koriyama, Japan), and midazolam (2.0 mg/kg; Sand, Tokyo, Japan) after aortical perfusion with cold saline. The collected tissues were stored at − 80 °C until use in the assay. The tissues stored at − 80 °C were thawed and homogenized in RIPA buffer (Nacalai Tesque, Kyoto, Japan) with a protease inhibitor (1:100; Takara, Otsu, Japan). Following centrifugation, the supernatants were collected, and total protein concentrations were measured using a BCA Protein Assay Kit (Thermo Fisher Scientific) or a bicinchoninic acid assay kit (Takara). Arterial blood samples were transcardially collected at P14, P28, and week 7 (11:00 a.m. to 2:00 p.m.) and centrifuged for 15 min at 3000 rpm and 4 °C. The collected serum was stored at − 80 °C until use in the assay.

### Measurement of plasma CORT level

To assess CORT levels at P14 and P28, their levels in collected plasma were measured using a CORT-specific enzyme-linked immunosorbent assay kit (YK-240; Yanaihara, Fujinomiya, Japan) according to the manufacturer’s instructions. Absorbance was measured at 450 nm using a spectrophotometer (Bio-Rad Laboratories Inc, Hercules, CA, USA). The absorbance values of the standards and samples were corrected by subtracting the background value to correct for absorbance attributable to nonspecific binding.

### Measurement of antioxidant capacity and mitochondrial complex I

Total antioxidant capacity in plasma was assessed using the OxiSelect™ Ferric Reducing Antioxidant Power Assay Kit (STA-859; Cell Biolabs, San Diego, CA, USA) according to the manufacturer’s protocol.

To measure mitochondrial complex I levels in the whisker pad skin, the homogenized tissue was assessed using the Complex I Enzyme Activity Assay Kit (ab109721; Abcam, Cambridge, UK). Briefly, 50-μg tissue samples were loaded into a 96-well plate, and enzyme activity was determined colorimetrically by detecting each sample’s light absorption value at 450 nm.

### Western blotting

Proteins (10 µg) were mixed with 2 × Laemmli buffer supplemented with 2-mercaptoethanol, and denatured for 5 min at 95 °C. Samples were loaded in a 4–20% Mini-PROTEAN TGX Precast Gel (Bio-Rad). After electrophoresis, proteins were transferred onto polyvinylidene difluoride membrane (Trans-Blot Turbo Transfer Pack, Bio-Rad). Membranes were blocked with TBST (0.2% Tween-20 diluted in Tris-buffered saline) containing 5% Blocking-One (Nacalai Tesque, Kyoto, Japan) for 1 h at room temperature and then incubated overnight at 4 °C with primary antibodies for anti-TRPA1 (rabbit, Polyclonal, 1:500; NB110-40763, Novus Biologicals, LLC, Centennial, CO, USA), and anti-β-actin antibody (mouse monoclonal, 1:200; sc-69879; Santa Cruz, San Diego, CA, USA). After washing with TBST 4 times for 5 min, membranes were incubated with horseradish peroxidase (HRP)-conjugated secondary antibodies for anti-rabbit IgG or anti-mouse IgG (1:2000, NA934V or NA931V; Cytiva, Marlborough, MA, USA) 2 h at room temperature. The bands were visualized using an image analyzer (Amersham Image Quant 800, Cytiva) after reaction in Western Lightning ELC Pro (PerkinElmer, Waltham, MA, USA) or Immobilon (Millipore, Burlington, MA, USA). The band intensity was quantified using ImageJ and normalized to β-actin expression.

### Immunohistochemistry

To evaluate oxidative stress and TRPA1 expression in the whisker pad skin, immunohistochemistry was performed using an anti-8-OHdG antibody, a marker of oxidative DNA damage, and an anti-TRPA1 antibody in whisker pad skin sections^37^. The whisker pad skin was removed and fixed overnight at 4 °C in 4% paraformaldehyde under deep anesthesia using the combination of anesthetics described above. The whisker pad skin tissue was sectioned to a thickness of 20 μm using a cryostat (CM1900; Leica, Wetzlar, Germany) and mounted on magnesium aluminosilicate (MAS)-coated glass (Matsunami, Osaka, Japan). Some 10-μm-thick sections were stained with hematoxylin and eosin. The sections were subsequently incubated with mouse monoclonal anti-8-OHdG antibody (MOG-100P, 10 μg/mL; Japan Institute for the Control of Aging, Fukuroi, Japan) or rabbit anti-TRPA1 antibody (NB110-40763, 5 μg/mL; Novus Biologicals) overnight at 4 °C after blocking with 3% normal goat serum for 1.5 h at 23–26 °C (room temperature). After incubation with 0.3% H_2_O_2_ for 30 min, the sections were incubated with biotinylated secondary antibodies, namely, anti-mouse IgG (BA2000, 1:600; Vector, Newark, CA, USA) or anti-rabbit IgG (BA1000, 1:600; Vector), for 2 h as well as avidin and biotinylated horseradish peroxidase complex (Vectastain ABC Kit; Vector) for 1 h at room temperature. The sections were immediately rinsed with phosphate-buffered saline after reacting with 0.05% diaminobenzidine dihydrochloride. As a negative control, the same immunohistochemical procedure was performed; however, the primary antibody was omitted. Hematoxylin was used to counterstain TRPA1 immunoreactivity. The sections were dehydrated in alcohol/xylene and covered with coverslips. The immunoreactivity for 8-OHdG was quantified by using ImageJ. The mean intensity of region in the whisker pad skin including epithelium (400 × 400 μm) was calculated in 3 sections of each rat.

### Statistical analysis

Data are expressed as the median and interquartile range (25–75%) or mean ± standard error of the mean. In the box-and-whisker plots, the upper and lower whiskers represent the maximum and minimum values, respectively, and the dotted plots indicate individual sample sizes, where N represents the number of rats tested. Data normality was confirmed using the Shapiro–Wilk test. The Mann–Whitney U test, as a non-parametric procedure for analyzing the mechanical withdrawal threshold, was used to compare the two groups. An unpaired *t*-test was used to compare body weight, 8-OHdG intensity, antioxidant capacity, complex I activity, amount of TRPA1, and CORT concentration between the two groups. Statistical significance was set at *P* < 0.05. Statistical analyses were performed using GraphPad Prism 8 software (GraphPad Prism Software, San Diego, CA, USA).

### Supplementary Information

Supplementary Information.

## Data Availability

The data that support the findings of this study are available from the corresponding author, S.H., upon reasonable request.
