# Supplementary material for: Involvement of oxidative stress in orofacial mechanical pain hypersensitivity following neonatal maternal separation in rats
Source: Sci Rep. 2023 Dec 20;13:22760. doi: 10.1038/s41598-023-50116-1 (PMC10733350; doi:10.1038/s41598-023-50116-1)
Supplement: Supplementary file 1 — Supplementary Information. [file 41598_2023_50116_MOESM1_ESM.docx]

**Involvement of oxidative stress in orofacial mechanical pain hypersensitivity following neonatal maternal separation in rats**

Chihiro Soma, Suzuro Hitomi, Eri Oshima, Yoshinori Hayashi, Kumi Soma, Ikuko Shibuta, Yoshiyuki Tsuboi, Tetsuo Shirakawa, Takashi Kikuiri, Koichi Iwata, Masamichi Shinoda

**Supplementary Table S1**. The number of rats used in the present study.

Pregnant rat # Male pup Female pup Total number

(Mother) used births used births of births

#1 8 8 4 4 12

#2 5 5 - 8 13

#3 7 7 - 8 15

#4 7 7 - 8 15

#5 6 6 - 3 9

#6 7 7 7 7 14

#7 6 6 6 6 12

#8 8 9 7 7 16

#9 8 8 4 4 12

#10 8 11 - 3 14

#11 2 4 - 10 14

#12 9 10 - 2 12

#13 6 7 - 5 12

#14 9 9 - 5 14

#15 5 6 - 9 15

#16 5 5 - 9 14

#17 8 8 - 7 15

#18 4 5 - 10 15

#19 4 7 - 8 15

#20 4 6 - 5 11

#21 4 7 - 5 12

#22 5 7 - 6 13

#23 6 6 - 7 13


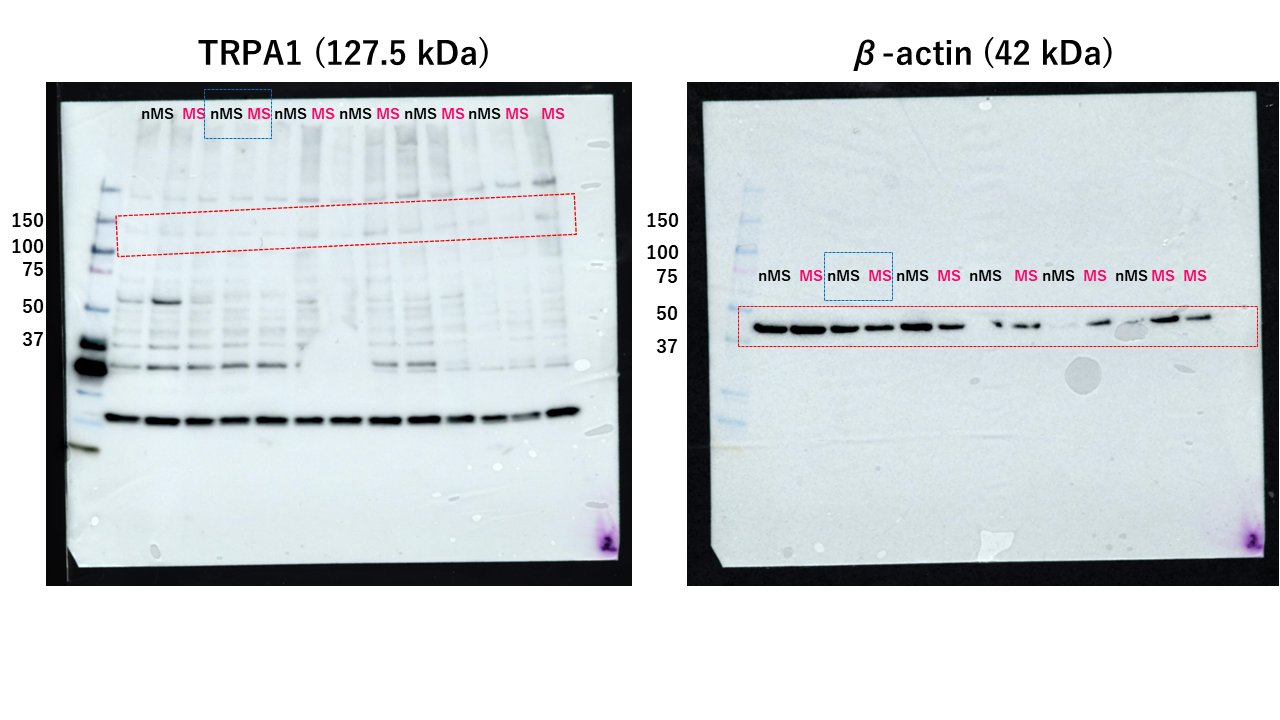
**Supplementary Figure S1**. Original images of western blotting.
